# Identification of novel chemotherapeutic strategies for metastatic uveal melanoma

**DOI:** 10.1038/srep44564

**Published:** 2017-03-17

**Authors:** Paolo Fagone, Rosario Caltabiano, Andrea Russo, Gabriella Lupo, Carmelina Daniela Anfuso, Maria Sofia Basile, Antonio Longo, Ferdinando Nicoletti, Rocco De Pasquale, Massimo Libra, Michele Reibaldi

**Affiliations:** 1 Department of Biomedical and Biotechnological Sciences, University of Catania, Catania, Italy; 2 Department of Medical and Surgical Sciences and Advanced Technologies, G.F. Ingrassia, University of Catania, Catania, Italy; 3 Department of Ophthalmology, University of Catania, Catania, Italy; 4 Department of Dermatology, AOU Policlinico Vittorio Emanuele Hospital Catania, Catania, Italy

## Abstract

Melanoma of the uveal tract accounts for approximately 5% of all melanomas and represents the most common primary intraocular malignancy. Despite improvements in diagnosis and more effective local therapies for primary cancer, the rate of metastatic death has not changed in the past forty years. In the present study, we made use of bioinformatics to analyze the data obtained from three public available microarray datasets on uveal melanoma in an attempt to identify novel putative chemotherapeutic options for the liver metastatic disease. We have first carried out a meta-analysis of publicly available whole-genome datasets, that included data from 132 patients, comparing metastatic vs. non metastatic uveal melanomas, in order to identify the most relevant genes characterizing the spreading of tumor to the liver. Subsequently, the L1000CDS^2^ web-based utility was used to predict small molecules and drugs targeting the metastatic uveal melanoma gene signature. The most promising drugs were found to be Cinnarizine, an anti-histaminic drug used for motion sickness, Digitoxigenin, a precursor of cardiac glycosides, and Clofazimine, a fat-soluble iminophenazine used in leprosy. *In vitro* and *in vivo* validation studies will be needed to confirm the efficacy of these molecules for the prevention and treatment of metastatic uveal melanoma.

Uveal melanoma is the most common primary intraocular cancer, and after the skin, the uveal tract is the second most common location for melanoma^1^. However, cutaneous and uveal melanomas are different in terms of biology, natural history and response to chemotherapies^2^.

The 5-year survival rate is 50–70% and about 50% of patients develop metastases within a median of 36 months, almost exclusively to the liver, with a median survival of 6 months after metastases^3^.

The most frequent chromosomal abnormalities in uveal melanoma are loss of chromosome 3 and gains of 8q and 6p. Patients with chromosome 3 loss undergo the worst prognosis, whereas those with chromosome 6 (6p) gain have the best outcomes. Mutations in G-protein-α subunits GNAQ or GNA11 are observed in ≥80% of primary uveal melanomas and inactivating BAP1 mutations are found in approximately 50% of all cases, most frequently in metastatic disease. Mutations that are associated with a less aggressive behavior are those in splicing factor 3B subunit 1 (SF3B1) and eukaryotic translation initiation factor 1A, X-linked (EIF1AX)^4^.

Local radiation therapy, using brachytherapy or alternatively, charged-particle and proton-beam radiation, is the most common approach^4^,^5^,^6^. Enucleation remains the only option for very large tumors.

Despite improved understanding of the disease, the overall survival (OS) rate has not increased since the 1970 s. Indeed, once uveal melanoma has metastasized to distant organs, the disease is resistant to current chemotherapies. Distant metastasis are infrequent at the time of initial presentation, occurring in <5% of patients. For patients who develop metastasis, there is yet no standard of care. Dacarbazine, has been used for uveal melanoma, but efficacy is limited. Other chemotherapeutics, i.e. temozolomide, cisplatin, treosulfan, fotemustine and various combinations have been tested in uveal melanoma with disappointing results. A few adjuvant studies have been performed in uveal melanoma to prevent metastatic disease. However, no significant effects on metastasis free survival or OS benefit have been obtained, up to date^4^.

In the present study, we made use of bioinformatics to analyze the data obtained from three public available microarray datasets on uveal melanoma in attempt to identify novel putative chemotherapeutic options for the metastatic disease.

## Material and Methods

### Microarray dataset selection and meta-analysis

The NCBI Gene Expression Omnibus (GEO) database (http://www.ncbi.nlm.nih.gov/geo/)^7^ was used to identify suitable microarray datasets comparing localized vs. metastatic uveal melanomas. Three datasets were included in the study GSE22138^8^- GSE73652^9^, and GSE44295, that included whole-genome transcriptomic data from primary melanoma cells obtained upon eye enucleation (Table 1). The following information were extracted from each of the studies that were selected: GEO accession; sample type; platform; numbers of patients and controls; and gene expression data. Briefly, the GSE22138 dataset included 28 samples of non-metastatic tumor and 35 samples from enucleated patients having distant metastasis. Patients were included in the group of localized tumors if no metastasis were observed in 36 months of follow-up. The GSE73652 dataset included 5 samples of non-metastatic tumor and 8 samples from enucleated patients having distant metastasis. Metastasis-free cases were included only if there was >6 years follow-up. No detailed clinical data are available for GSE44292, that included 32 samples from patients with localized disease and 24 sample from patients with metastasis. Illumina platforms were pre-processed using Bead-array prior to quantile normalization (Dunning *et al*., 2007), while Affymetrix platforms were pre-processed and quantile normalized using the robust multiarray average (RMA). The datasets were uploaded to INMEX (http://www.inmex.ca/INMEX)^10^, and the data annotated by converting probe ID to Entrez IDs. For each probe-set, intensity values were auto-scaled and a data integrity check was performed prior to the meta-analysis stage. Batch effects were corrected using the “ComBat” function. A random effects model of effect size (ES) measure was used to integrate gene expression patterns from the three datasets. The random effects model presumes that different studies present substantial diversity, and evaluates between-study variance as well as within-study sampling error. Genes with FDR < 0.01 were identified as Differentially Expressed (DE) and selected for further analysis.

### Gene Ontology (GO) and Drug Prediction Analysis

Functional relationships among DE gene were obtained from GeneMania (http://genemania.org/ http://genemania.org/)^11^. GeneMANIA searches publicly available genomics and proteomics data, including data from gene and protein expression profiling studies and primary and curated molecular interaction networks and pathways, to find related genes. The network weighting method is ‘Gene-Ontology (GO) based weighting, Biological Process based’. This weighting method assumes the input gene list is related in terms of biological processes (as defined by GO).

The PANTHER (protein annotation through evolutionary relationship) classification system^12^ (http://www.pantherdb.org/) was used to classify input genes according to their function (PANTHER protein class). PANTHER is a comprehensive system that combines gene function, ontology, pathways and statistical tools that enable to analyze large-scale, genome-wide data from sequencing, proteomics or gene expression experiments.

The L1000CDS^2^ was used to identify potential chemotherapeutics for metastatic uveal melanoma. L1000CDS^2^ enables to find L1000 small molecule signatures that match input gene signatures. The L1000 mRNA gene-expression dataset is generated as part of the Library of Integrated Network-based Cellular Signatures (LINCS) project, a NIH Common Fund program. LINCS aims to profile the molecular effects of small molecules on human cells^13^. When gene lists are submitted to L1000CDS^2^, the search engine compares the input gene lists to the DE genes computed from the LINCS L1000 data and returns the top 50 matched signatures. The result score is the overlap between the input DE genes and the signature DE genes divided by the effective input. The effective input is the length of the intersection between the input genes and the L1000 genes. L1000CDS^2^ currently covers chemically perturbed gene expression profiles from 62 cell-lines and 3,924 small molecules. Also, L1000CDS^2^ allows to predict effective drug combinations by comparing every possible pair among the top 50 signatures and computing the potential synergy for each pair.

## Results

### Meta-analysis of gene expression in metastatic uveal melanoma

**Figure 1:**
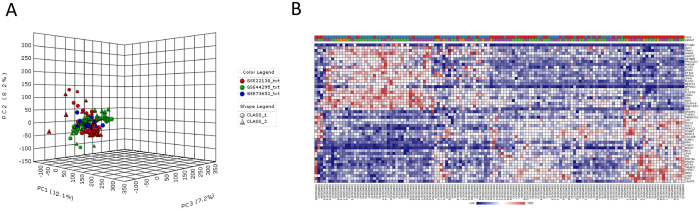
(**A**) Principal Component analysis of GSE73652, GSE44295 and GSE22138. CLASS1 refers to metastatic tumors and CLASS2 to localized tumor; (**B**) Heatmap showing the 64 differentially expressed genes as determined by Random Effect Size Analysis of GSE73652, GSE44295 and GSE22138.

Three GEO data sets were identified for the following analysis (Table 1). These datasets consisted of primary uveal melanoma data, and included a total of 67 metastatic tumors and 65 localized primary tumors. Details of the individual studies are presented in Table 1. Figure 1A shows a Principal Component Analysis of the three separate microarray datasets included in the meta-analysis.

**Figure 2:**
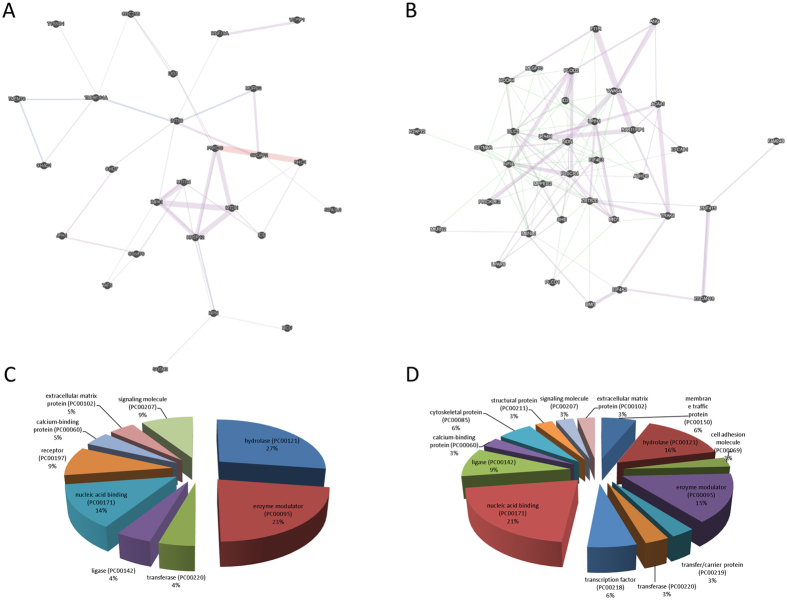
(**A**) Functional network of the significant upregulated genes obtained from the meta-analysis of GSE73652, GSE44295 and GSE22138; (**B**) Functional network of the significant downregulated genes obtained from the meta-analysis of GSE73652, GSE44295 and GSE22138; (**C**) Protein class analysis for the significant upregulated genes obtained from the meta-analysis of GSE73652, GSE44295 and GSE22138; (**D**) Protein class analysis for the significant downregulated genes obtained from the meta-analysis of GSE73652, GSE44295 and GSE22138.

A total of 64 genes were identified, which were consistently modulated in metastatic tumors. Among these 64 DE genes, 29 were upregulated and 35 were downregulated. A list of the significant upregulated and downregulated genes is shown in Tables 2 and 3, respectively. An heatmap showing the 64 DE genes is presented in Fig. 1B. The functional relationships among the up- and down-regulated genes, respectively, are presented in Fig. 2A,B. Among the upregulated genes, the top three represented protein classes were: Transferase, which included phosphatases (CDC25B and IMPA1) and proteases (IDE and TYSND1); Enzyme Modulator, including G protein modulators (SGSM2, SRGAP2 and SIPA1L2) and the protease inhibitor, HPN; Nucleic Acid Binding, i.e. the DNA helicase, CHD7, and the DNA ligase, LIG1 (Fig. 2C).

Among the downregulated genes, the top three protein classes represented were: Nucleic Acid Binding, that included DNA binding proteins (H2AFY2, NDN and MBNL1) and RNA binding proteins (ANG, EIF4A2 and EIF4A3); Hydrolase, including the proteases, ABHD6 and LTA4H, and the lipase, PLCD1; Enzyme Modulator, including the G protein modulator, PLCD1, the kinase modulator, HOOK1, and the protease inhibitor, VWA5A (Fig. 2D).

### Prediction of novel chemotherapeutics for metastatic uveal melanoma

**Figure 3:**
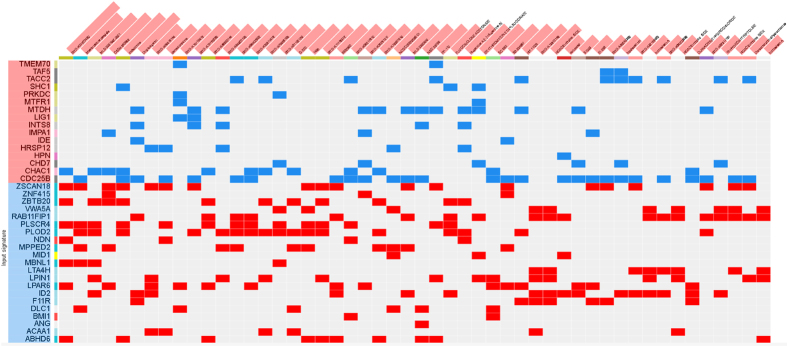
Clustergram with the top ranked L1000 perturbations (e.g. those with most anti-similar signatures). Input genes (rows) are represented by the significantly upregulated (highlighted in red) and downregulated genes (highlighted in blu) obtained from the meta-analysis of GSE73652, GSE44295 and GSE22138. Columns include the top predicted drugs targeting input genes.

The L1000CDS^2^ web-based utility was used to predict small molecules and drugs targeting the metastatic uveal melanoma gene signature. Figure 3 shows a clustergram with the top ranked L1000 perturbations (e.g. those with most anti-similar signatures). The complete list of predicted chemotherapeutics is presented as Table 4. Several of these drugs are FDA-approved and already used in the clinic or tested in clinical trials, as indicated in Table 4. Among the predicted chemotherapeutics, the most represented classes were: histone deacetylase inhibitors (that included HDAC6 inhibitor ISOX, BRD-K13810148, Trichostatin A and Vorinostat), and anti-infectious/parasitic drugs (i.e., Clofazimine, Erythromycin ethylsuccinate, Demeclocycline and Quinacrine hydrochloride). The top identified drugs were the following: BRD-K07220430 (Cinnarizine), an anti-histaminic drug used for motion sickness, was predicted for its ability to downregulate CHAC1 and to upregulate MBNL1, LPAR6, PLSCR4, NDN, ABHD6, ZSCAN18 and ZBTB20; Digitoxigenin, for its ability to downregulate CDC25B, IDE, INTS8 and MTDH, while upregulating F11R, ID2, and RAB11FIP1; clofazimine, a fat-soluble iminophenazine used in leprosy, which is able to downregulate CDC25B, CHAC1, and SHC1, and to upregulate ABHD6, PLOD2, PLSCR4, ZBTB20, ZSCAN18. Accordingly, the top three most promising drug combination found were: BRD-K07220430 and Digitoxigenin; Digitoxigenin and OSSK_645683; BRD-K07220430 and HDAC6 inhibitor ISOX (for the complete list, see Table 5).

## Discussion

Melanoma of the uveal tract accounts for 5% of all melanomas and, with an incidence of about 6 cases per million person–years, represents the most common primary intraocular malignancy^14^,^15^. Despite improvements in diagnosis and more effective local therapies for primary cancer, the rate of metastatic death has not changed in the past forty years^15^.

Several genes and pathways have been identified to be involved in the progression and metastasis of uveal melanoma, such as c-Met^16^, Hepatocyte Growth Factor (HGF)^16^,^17^, Insulin-like Growth Factor-1 Receptor (IGF-1R)^18^, the CXCL12-CXCR4 pathway^17^,^19^, VEGF^20^, Mda-9/syntenin^21^ and the PTP4A3 phosphatase^8^. However, despite increasing knowledge in the biology of uveal melanoma, no therapeutic strategies has been found to be effective. Metastatic uveal melanoma is resistant to current systemic chemotherapy and, to date, no clear role has been established for chemotherapy in several clinical trials, that reported objective response rates <20%^4^. Single-agent chemotherapies (e.g., dacarbazina, fotemustine, paclitaxel, temozolomide, camptothecin, treosulfan) or combination chemotherapies (e.g. gemcitabine/treosulfan, cisplatin/gemcitabine/treosulfan, carboplatin/paclitaxel/sorafenib) have shown poor response rates and immunotherapy with ipilimumab, antiangiogenetic treatment strategies using bevacizumab combined with interferon-α2b, temozolomide, or aflibercept have not proven to be superior to chemotherapy^22^.

In order to prevent metastatic disease, several adjuvant studies are also being conducted using ipilimumab, sunitinib, valproic acid, and crizotinib for high-risk patients^4^. Adjuvant treatment with bacillus Calmette-Guerin and interferon-α, as well as, intra-arterial hepatic infusion of fotemustine, have been previously studied in an effort to reduce the occurrence of liver metastasis, but none of these studies has demonstrated significant improvement in metastasis free survival or OS^4^.

The limited efficacy of current chemotherapies proves the medical need for more effective treatment strategies in metastatic uveal melanoma. In the present study, a meta-analysis of three whole-genome microarray datasets on primary tumor samples from the enucleated eyes of patients with localized or metastatic disease, has been performed in order to investigate the genes associated to the metastatic properties of uveal melanoma and to identify potential chemotherapeutic strategies to be used in metastatic disease.

It was recently developed a 15-gene qPCR-based assay that discriminate between primary uveal melanomas that have a low metastatic risk and a high metastatic risk 23. This platform is currently used in a College of American Pathologists (CAP)-accredited Clinical Laboratory Improvement Amendments (CLIA)-certified laboratory on fine needle aspiration samples and on formalin-fixed specimens^23^. A few of the genes screened in this assay correspond to those identified by the current analysis, namely ID2 and LTA4H, among the downregulated genes. Some of the other DE genes, share similar function to those included in the assay, i.e. EIF4A2 and EIF4E2 (to EIF1B).

As regards the putative drugs here identified, some of them are already in clinical use, such as BRD-K07220430 (Cinnarizine)^24^, clofazimine^25^, mesoridazine besylate^26^, erythromycin ethylsuccinate^27^; other are in preclinical development (e.g. HDAC6 inhibitor ISOX)^28^ or do not have any known pharmacological target, e.g. OSSK_645683. Interestingly, some of the identified drugs are histone deacetylase inhibitors (such as, HDAC6 inhibitor ISOX, BRD-K13810148, Trichostatin A and Vorinostat), that have already been shown efficacy on uveal melanoma preclinical models^29^,^30^,^31^,^32^,^33^,^34^,^35^,^36^. Klisovic and collaborators have shown that the histone deacetylase inhibitor, Depsipeptide (FR901228), inhibits proliferation and induces apoptosis in primary and metastatic human uveal melanoma cell lines^35^, as well as it is able to inhibit *in vitro* uveal melanoma cell lines migration via downregulation of Matrix MetalloProteinases 2, 9 and Membrane Type-1/MMP (MMP-2, MMP-9 and MT-1/MMP) and the upregulation of Tissue Inhibitors of Matrix MetalloProteinases 1 and 2 (TIMP-1 and TIMP-2)^34^. Chen and collaborators have shown that microRNA-137 and microRNA-124a act as a tumor suppressors in uveal melanoma and could be successfully silenced by using the histone deacetylase inhibitor, trichostatin A^32^,^36^. Landreville and collegues have shown that in three uveal melanoma cell lines (92.1, OCM1A, and Mel202), Trichostatin A was able to reduce the fraction of viable cells and increase the proportion of cells undergoing apoptosis^33^. Also, it was shown that Quisinostat (a Class I and II histone deacetylase inhibitor) inhibited the migration and proliferation of 92.1 and OMM2.3 cell lines in zebrafish xenograft embryos^30^. In addition, the Class III-specific HDAC inhibitor, Tenovin-6, was shown to be able to eradicate cancer stem cells in 92.1 and Mel 270 cells^29^. Venza in 2014^31^, reported that the Class-I histone deacetylase inhibitor, MS-275, due to its ability to reduce c-FLIP expression, is able to increase TRAIL-induced cell death in uveal melanoma cell lines. MS-275, is currently tested in a Phase 1 study for the treatment of patients with Refractory Solid Tumors, including intraocular melanoma, or Lymphomas (NCT00020579). Vorinostat, a small molecule inhibitor of class I and II histone deacetylases, is currently tested in two Phase 2 Study on Ocular Melanoma With Extraocular Extension, Recurrent Uveal Melanoma and Grade IV Metastatic Uveal Melanoma (NCT00121225, NCT 01587352).

The utility of the present data may reside primarily in the potential identification of effective adjuvant treatments. In light of the fact that there is no therapy for metastatic disease, the most promising strategy to improve survival is to treat patients in an adjuvant setting. Although there might be non- metastasizing melanomas, which never metastasize, even if not treated, however the intra-tumoral genetic heterogeneity suggests an ongoing evolutionary tumoral process^37^. Adjuvant interventions could settle up the uncertainty about the effect of ocular treatment on survival, since some patients might unnecessarily sacrifice their visus in the hope of a longer life-expectancy; while other patients with small melanomas might succumb due to metastasis because treatment has been delayed. Presently, there is no adjuvant approach that improves outcome, but the impact of ocular treatment, in terms of therapeutic benefit, has ethical implications.

The present study has several advantages but also limitations. It represents, to date, the largest meta-analysis comparing metastatic vs. non metastatic uveal melanomas, encompassing whole-genomic data from a total of 132 patients. A strict and rigorous statistics has been applied to data in order to sort out the most relevant genes characterizing the spreading of tumor to the liver. Among the upregulated genes, the highest effect size was detected for JPH1, encoding for Junctophilin-1, a component of junctional complexes between the plasma membrane and endoplasmic/sarcoplasmic reticulum, providing functional cross-talk between the cell surface and intracellular calcium release channels (http://www.genecards.org/cgi-bin/carddisp.pl?gene=JPH1). Among the significant downregulated genes, the gene with the highest effect size was PLSCR4, encoding for the Phospholipid Scramblase 4, which has been found to be strongly expressed in the neuropil of malignant gliomas and in cytoplasm of liver cancers, colorectal cancers and malignant melanomas (http://www.proteinatlas.org/ENSG00000114698-PLSCR4/cancer). Comparing to the original analysis, the PTP4A3 phosphatases and the cancer-testis antigen, PRAME, were dropped out because of too little statistical significance. In addition, although the role of the PKC, MAPK and PI3K/AKT/mTOR signaling cascades has been extensively examined in uveal melanoma^38^,^39^,^40^, however, whole genome transcriptomic analysis may fail to detect their modulation, because of the primary contribution related to post-transcriptional modifications. Also, our analysis was very statistical stringent (FDR < 0.01), and this may account for the differences from previously published work^1^. Furthermore, the discrepancies with previous analysis may be due to the enlargement of the number of samples which could influence DE genes detection as respect to smaller sample groups.

Investigating the genes found to be significantly modulated in the present analysis may help to identify the molecular mechanisms involved in the liver metastasis of uveal melanoma, and may allow the identification of novel pharmacological targets for the prevention and treatment of liver involvement. Moreover, our study was aimed at predicting putative pharmacological schemes to apply as adjuvant or curative therapy, making use of the Library of Integrated Network-based Cellular Signatures (LINCS) project database. The repurposing of drugs currently approved for use in the clinical setting may expedite the design of phase II-III clinical trials, being cost-effective and reducing the risks associated with early stages of drug development^41^. The highest ranking scores were found for Cinnarizine, Digitoxigenin and Clofazimine. Cinnarizine has already been shown to be effective *in vitro* against lymphoma and multiple myeloma^42^ and to inhibit melanogenesis in mouse B16 melanoma cells^43^, and Clofazimine was found effective in preclinical models of pancreatic ductal adenocarcinoma and triple-negative breast cancer^44^,^45^.

However, this study has also limitations. Despite the practicality of this bioinformatic approach, there are drawbacks to point out. First, the efficacy of a drug is more complex than the simple match of expression profiles. Drugs have to reach tissue-specific concentrations to exert an effect, and the route and timing of administration is essential for the drug to be effective and to limit side effects. A preliminary *in vitro* testing on both primary and established cancer cell lines will be required before running pilot phase II clinical trials. On the other hand, for successful result of the clinical trials, the appropriate selection and recruitment of patients will be also key to evaluate the potential activity of the compounds in the clinical setting.

## Additional Information

**How to cite this article:** Fagone, P. *et al*. Identification of novel chemotherapeutic strategies for metastatic uveal melanoma. *Sci. Rep.* **7**, 44564; doi: 10.1038/srep44564 (2017).

**Publisher's note:** Springer Nature remains neutral with regard to jurisdictional claims in published maps and institutional affiliations.

## Figures and Tables

**Table 1:** Characteristics of the microarray datasets included in the meta-analysis.

Study	GEO accession	Patients		Samples	Platform
		Localized (n.)	Metastatic (n.)		
1	GSE22138	28 Mean age: 59.1 Chr3 monosomy: 10/28; Partial alteration: 2/28; Not available:2/28 36 months follow-up	35 Mean age: 60.5 Chr3 monosomy: 22/35; Partial alteration: 3/35; Not available: 6/35	Primary tumor (eye)	Affymetrix U133 plus 2
2	GSE44295	32	24	Primary tumor (eye)	Illumina Human ref. 8 v.3
3	GSE73652	5 > 6 years follow-up	8	Primary tumor (eye)	Illumina Human HT 12 v. 4

**Table 2:** List of significantly upregulated genes in metastatic uveal melanoma.

Name	Combined Effect Size	FDR
JPH1	−1.1736	1.5378e-05
TACC2	−0.97146	0.00059404
SIPA1L2	−0.96664	0.00059404
TYSND1	−0.9641	0.00059404
TMEM161A	−0.95064	0.003168
MCU	−0.94061	0.00094886
PRKDC	−0.93729	0.00094886
MTFR1	−0.90776	0.0015792
SHC1	−0.86702	0.003168
MYEOV	−0.86335	0.003168
VCPIP1	−0.85848	0.003168
CHAC1	−0.8543	0.003168
HPN	−0.85398	0.003168
SUMF2	−0.85012	0.0049617
LIG1	−0.83865	0.0040445
RNF19A	−0.83777	0.0040445
TAF5	−0.8377	0.0040445
HRSP12	−0.83574	0.009672
CDC25B	−0.83518	0.0040445
IMPA1	−0.82144	0.0048032
SGSM2	−0.81096	0.0052385
SRGAP2	−0.80528	0.0057402
MORC2	−0.79995	0.0057952
C8orf76	−0.79478	0.0059805
TMEM70	−0.79037	0.0063563
IDE	−0.78739	0.0065272
CHD7	−0.78327	0.0071536
MTDH	−0.7758	0.0082124
INTS8	−0.75952	0.009672

**Table 3:** List of significantly downregulated genes in metastatic uveal melanoma.

Name	Combined Effect Size	FDR
PLSCR4	1.1795	0.0072336
RAB11FIP1	1.0587	0.00013411
MEGF10	1.0539	0.00013411
ID2	1.0091	0.00046632
VWA5A	0.93182	0.005083
TRAK1	0.91734	0.0014595
MPPED2	0.90563	0.0015792
ANG	0.89382	0.0050995
DLC1	0.87069	0.003168
NDN	0.86139	0.003168
PLCD1	0.85619	0.003168
EFCAB1	0.8339	0.004092
ACAA1	0.8233	0.0048032
LPIN1	0.82127	0.0048032
LTA4H	0.81998	0.0048032
BMI1	0.80767	0.0086087
NFIA	0.80698	0.0057402
PRICKLE2	0.80144	0.0057952
H2AFY2	0.8013	0.0057952
SETMAR	0.79757	0.0059412
PLOD2	0.79556	0.0059805
EIF4A2	0.79517	0.0059805
FAM24B	0.79409	0.0059805
ZSCAN18	0.78983	0.0063563
MKRN2	0.78026	0.0071738
F11R	0.77016	0.0084361
HOOK1	0.76951	0.00849
EIF4E3	0.76619	0.0089451
LPAR6	0.76421	0.0092481
MID1	0.7632	0.0091958
ZBTB20	0.76021	0.009672
ABHD6	0.76011	0.009672
MBNL1	0.75836	0.009672
SHE	0.75664	0.0098953
ZNF415	0.75661	0.009672

**Table 4:** Predicted small molecules and drugs targeting the metastatic uveal melanoma gene signature.

Rank	Score	Perturbation	Class*	FDA status*	Indications*
1	0.1569	BRD-K07220430 (Cinnarizine)	Anti-histaminic	Approved	Motion sickness.
2	0.1373	Digitoxigenin	Unknown	Not approved	None
3	0.1373	clofazimine	Antimycobacterial	Approved	Leprosy
4	0.1373	OSSK_645683	Unknown	Not approved	None
5	0.1373	MLS-0091942.0001	Unknown	Not approved	None
6	0.1373	Mesoridazine besylate	Neuroleptic	Approved	Schizophrenia
7	0.1176	HDAC6 inhibitor ISOX	Histone deacetylase inhibitor	Not approved	None
8	0.1176	BRD-K13810148	Histone deacety-lase inhibitor	Investigational	
9	0.1176	HY-11005 (BX-912)	PDK1 inhibitor	Not approved	None
10	0.1176	SB 334867	Orexin antagonist	Not approved	None
11	0.1176	SC 560	Cyclooxygenase inhibitors	Not approved	None
12	0.1176	ERYTHROMYCIN ETHYLSUCCINATE	Macrolide antibiotic	Approved	Bacterial infection
13	0.1176	Huperzine A [(−)-Huperzine A]	Reversible acetylcholinesterase inhibitor	Investigational	Alzheimer’s disease
14	0.1176	FLUOCINOLONE ACETONIDE	Glucocorticoid	Approved	Skin disorders
15	0.1176	PP-110	Topical anesthetic	Investigational	Hemorrhoids
16	0.1176	NSC 23766	RAC1 inhibitor		
17	0.1176	MLS-0390979	Inhibitor of IAPs		
18	0.1176	NCGC00229596-01	Unknown	Not approved	None
19	0.1176	BRD-K73567619	Unknown	Not approved	None
20	0.1176	BRD-K24281017	Unknown	Not approved	None
21	0.1176	BRD-K06217810	Unknown	Not approved	None
22	0.1176	5922592	Unknown	Not approved	None
23	0.1176	BRD-K11778372	Unknown	Not approved	None
24	0.1176	1495	Unknown	Not approved	None
25	0.1176	G-220	Unknown	Not approved	None
26	0.1176	BRD-K61192129	Unknown	Not approved	None
27	0.1176	BRD-K75958195	Unknown	Not approved	None
28	0.1176	BRD-K50311478	Unknown	Not approved	None
29	0.1176	BRD-K96402602	Unknown	Not approved	None
30	0.1176	BRD-K94920105	Unknown	Not approved	None
31	0.1176	BRD-K48692744	Unknown	Not approved	None
32	0.1176	BRD-K12765235	Unknown	Not approved	None
33	0.1176	BRD-K15715913	Unknown	Not approved	None
34	0.1176	demeclocycline	Semisynthetic tetracycline antibiotic	Approved	Lyme disease, acne, and bronchitis
35	0.1176	BRD-K49519144	Unknown	Not approved	None
36	0.0980	trichostatin A	Histone deacety-lase inhibitor	Not approved	None
37	0.0980	Dorsomorphin dihydrochloride	AMPK inhibitor	Not approved	None
38	0.0980	HDAC6 inhibitor ISOX	Histone deacety-lase inhibitor	Not approved	None
39	0.0980	16-HYDROXYTRIPTOLIDE	Unknown	Not approved	None
40	0.0980	BRD-K92317137	Unknown	Not approved	None
41	0.0980	QUINACRINE HYDROCHLORIDE	Anti-helmintic	Approved	Giardiasis, cutaneous leishmaniasis, malignant effusions
42	0.0980	HDAC6 inhibitor ISOX	Histone deacety-lase inhibitor	Not approved	None
43	0.0980	BRD-K84203638		Not approved	None
44	0.0980	trichostatin A	Histone deacety-lase inhibitor	Not approved	None
45	0.0980	BRD-K92158425		Not approved	None
46	0.0980	topotecan hcl	DNA topoisomerases inhibitor	Approved	Cancer
47	0.0980	BRD-A60245366	Unknown	Not approved	None
48	0.0980	EI-293	Unknown	Not approved	None
49	0.0980	S1003	Unknown	Not approved	None
50	0.0980	vorinostat	Histone deacetylase inhibitors	Approved	Cutaneous T-cell lymphoma

^*^Information obtained from DrugBank (https://www.drugbank.ca/).

**Table 5:** Top 50 predicted small molecule combinations targeting the metastatic uveal melanoma gene signature.

Rank	Score	Combination	
1	0.2941	1. BRD-K07220430	2. Digitoxigenin
2	0.2745	2. Digitoxigenin	4. OSSK_645683
3	0.2745	1. BRD-K07220430	7. HDAC6 inhibitor ISOX
4	0.2745	1. BRD-K07220430	8. BRD-K13810148
5	0.2745	1. BRD-K07220430	12. ERYTHROMYCIN ETHYLSUCCINATE
6	0.2745	1. BRD-K07220430	18. NCGC00229596-01
7	0.2745	1. BRD-K07220430	30. BRD-K94920105
8	0.2549	2. Digitoxigenin	3. clofazimine
9	0.2549	2. Digitoxigenin	5. MLS-0091942.0001
10	0.2549	2. Digitoxigenin	6. Mesoridazine besylate
11	0.2549	3. clofazimine	7. HDAC6 inhibitor ISOX
12	0.2549	4. OSSK_645683	7. HDAC6 inhibitor ISOX
13	0.2549	6. Mesoridazine besylate	7. HDAC6 inhibitor ISOX
14	0.2549	3. clofazimine	8. BRD-K13810148
15	0.2549	4. OSSK_645683	8. BRD-K13810148
16	0.2549	6. Mesoridazine besylate	8. BRD-K13810148
17	0.2549	1. BRD-K07220430	9. HY-11005
18	0.2549	5. MLS-0091942.0001	10. SB 334867
19	0.2549	4. OSSK_645683	12. ERYTHROMYCIN ETHYLSUCCINATE
20	0.2549	6. Mesoridazine besylate	12. ERYTHROMYCIN ETHYLSUCCINATE
21	0.2549	1. BRD-K07220430	16. NSC 23766
22	0.2549	4. OSSK_645683	16. NSC 23766
23	0.2549	5. MLS-0091942.0001	16. NSC 23766
24	0.2549	1. BRD-K07220430	17. MLS-0390979
25	0.2549	5. MLS-0091942.0001	18. NCGC00229596-01
26	0.2549	1. BRD-K07220430	20. BRD-K24281017
27	0.2549	5. MLS-0091942.0001	20. BRD-K24281017
28	0.2549	2. Digitoxigenin	23. BRD-K11778372
29	0.2549	2. Digitoxigenin	24. 1495
30	0.2549	2. Digitoxigenin	25. G-220
31	0.2549	2. Digitoxigenin	26. BRD-K61192129
32	0.2549	4. OSSK_645683	26. BRD-K61192129
33	0.2549	2. Digitoxigenin	27. BRD-K75958195
34	0.2549	1. BRD-K07220430	29. BRD-K96402602
35	0.2549	1. BRD-K07220430	32. BRD-K12765235
36	0.2549	1. BRD-K07220430	33. BRD-K15715913
37	0.2549	2. Digitoxigenin	33. BRD-K15715913
38	0.2549	3. clofazimine	33. BRD-K15715913
39	0.2549	5. MLS-0091942.0001	33. BRD-K15715913
40	0.2549	1. BRD-K07220430	39. 16-HYDROXYTRIPTOLIDE
41	0.2549	1. BRD-K07220430	42. HDAC6 inhibitor ISOX
42	0.2549	1. BRD-K07220430	43. BRD-K84203638
43	0.2549	1. BRD-K07220430	44. trichostatin A
44	0.2549	1. BRD-K07220430	46. topotecan hcl
45	0.2549	1. BRD-K07220430	50. vorinostat
46	0.2353	4. OSSK_645683	5. MLS-0091942.0001
47	0.2353	1. BRD-K07220430	6. Mesoridazine besylate
48	0.2353	4. OSSK_645683	6. Mesoridazine besylate
49	0.2353	4. OSSK_645683	9. HY-11005
50	0.2353	5. MLS-0091942.0001	9. HY-11005
